# Short-Term Outcomes of Augmentation Urethroplasty in Recurrent Urethral Stricture Following Repeated Failed Optical Internal Urethrotomy: A Prospective Study

**DOI:** 10.7759/cureus.110857

**Published:** 2026-06-14

**Authors:** Utkarsh Bhandari, Sagar Singh, Harsh Darshan, Pratyush Kaithwas, Deepak K Nayak, Ashish Tiwari

**Affiliations:** 1 Urology, Gandhi Medical College, Bhopal, IND; 2 General Surgery, Gandhi Medical College, Bhopal, IND

**Keywords:** augmentation urethroplasty, buccal mucosal graft, clavien-dindo classification, internal optical urethrotomy, international prostate symptoms score (ipss), urethral reconstruction, urethral stricture disease, uroflowmetry outcomes

## Abstract

Introduction

Recurrent urethral stricture following failed optical internal urethrotomy (OIU) is a clinically challenging condition with limited prospective outcome data from South Asian tertiary care centres. Augmentation urethroplasty using buccal mucosal graft (BMG) is increasingly employed in this setting; however, its outcomes in patients with prior failed endoscopic management remain poorly characterised in the Indian literature.

Methods

This prospective study enrolled 20 male patients with recurrent urethral stricture following a minimum of two failed OIU procedures at a tertiary care centre in central India between September 2022 and September 2024. Pre- and post-operative International Prostate Symptom Score (IPSS) and maximum urinary flow rate (Qmax) were recorded at one, three, and six months. Postoperative complications were graded using the Clavien-Dindo classification. Success was defined as Qmax ≥15 ml/second at six months without urethral re-intervention. Statistical analysis used the Wilcoxon signed-rank test, the Friedman test, and the one-sample Wilcoxon signed-rank test for Qmax assessment.

Results

Mean preoperative IPSS was 17.90 ± 1.86, falling to 3.33 ± 1.19 at six months (p<0.001, Wilcoxon signed-rank test). All patients had a preoperative Qmax below 10 ml/second; mean postoperative Qmax was 17.33 ± 2.14 ml/second at six months, representing a statistically significant improvement from baseline (p<0.001). The success rate at six months was 94.4% (17/18). BMG was used in 85% of patients; dorsal onlay was the predominant technique (80%). Major complications (Clavien-Dindo Grade 3) occurred in 10% of patients; 90% experienced no or minor complications.

Conclusions

BMG augmentation urethroplasty achieves high short-term success rates with significant symptomatic improvement and an acceptable complication profile in recurrent urethral stricture following failed OIU, supporting its role as the procedure of choice after failed endoscopic management.

## Introduction

Male urethral stricture disease is a clinically significant condition with a reported incidence of 0.6-1.2% in developed nations and likely higher in South Asian countries where infective aetiologies remain prevalent [1,2]. The clinical burden includes lower urinary tract symptoms, recurrent urinary tract infections, and, in advanced cases, upper tract deterioration and renal insufficiency [3,4].

Optical internal urethrotomy (OIU) is among the most commonly performed endoscopic procedures for urethral stricture disease. However, its long-term success rate is limited, with recurrence rates ranging from 23% to 92% after a single procedure, approaching 100% following multiple attempts [5,6]. Risk factors for recurrence include stricture length greater than 2 cm, dense spongiofibrosis, and prior failed endoscopic interventions [7,8].

In patients who have failed OIU, open urethroplasty is considered the gold standard for definitive management [9]. Augmentation urethroplasty using buccal mucosal graft (BMG) has emerged as the technique of choice for anterior urethral strictures, owing to the favourable tissue characteristics of oral mucosa, including its hairless surface, rich submucosal vascular plexus, and resilience in a moist environment [10,11]. Both dorsal and ventral onlay configurations have been described, with technique selection guided by stricture location, urethral plate quality, and surgeon experience [12].

Despite widespread adoption of BMG urethroplasty, prospective outcome data specifically examining patients with recurrent stricture following repeated failed OIU remain limited. The present study was designed to prospectively evaluate the short-term functional outcomes, symptomatic improvement, and complication profile of BMG augmentation urethroplasty in this patient cohort. The primary outcome was defined as a Qmax of ≥15 ml/second at six months without the need for urethral re-intervention.

## Materials and methods

This was a prospective, single-arm, observational study conducted at the Department of General Surgery, Gandhi Medical College and Hamidia Hospital, Bhopal, Madhya Pradesh, India, from September 2022 to September 2024. Ethical clearance was obtained from the Institutional Ethics Committee (IEC) of Gandhi Medical College, Bhopal (letter number: 32313/MC/IEC/2022). The study was conducted in accordance with the Declaration of Helsinki and its amendments. Written informed consent was obtained from all participants prior to enrolment.

Study population

Inclusion criteria were: adult male patients presenting with recurrent urethral stricture following a minimum of two prior failed OIU procedures, diagnosis confirmed by retrograde urethrogram (RGU), uroflowmetry, and cystoscopy, preoperative Qmax below 10 ml/second, and willingness to undergo surgical intervention and comply with follow-up.

Exclusion criteria were: refusal of consent, active urinary tract infection (positive urine culture at time of surgery), primary post-traumatic urethral stricture (pelvic fracture urethral injury), neurogenic bladder dysfunction, prior urethroplasty (previous open urethral reconstruction), malignant urethral stricture, and posterior urethral pathology.

No a priori sample size calculation was performed; the sample of 20 patients represents a convenience sample based on the number of eligible patients presenting during the study period who met strict inclusion criteria.

Preoperative assessment

All patients underwent standardised preoperative evaluation including complete blood count, renal function tests, blood glucose, HIV serology, urine culture, and abdominal ultrasonography. Stricture characteristics were assessed by RGU and flexible cystoscopy. The International Prostate Symptom Score (IPSS) [13] was recorded for all patients. Patients with ultrasonographic evidence of prostatomegaly were commenced on alpha-blockers preoperatively.

Surgical technique

All procedures were performed under general anaesthesia in the lithotomy position by qualified surgeons with experience in urethral reconstruction. Prophylactic intravenous antibiotics (third-generation cephalosporin or ampicillin-gentamicin combination) were administered at induction. The decision to use dorsal or ventral onlay configuration was made intraoperatively based on urethral plate quality, lumen calibre at the stricture site, and accessibility of the dorsal urethral surface. Dorsal onlay was selected when the urethral plate was considered adequate for tubularisation, defined as a plate width sufficient to accommodate a 16Fr catheter without tension; ventral onlay was employed when ventral spongiosum quality was superior or when dorsal dissection posed unacceptable risk. While no formally validated decision algorithm was applied, intraoperative decisions were consistent with published principles of urethral plate assessment described by Barbagli et al. [12].

BMG was harvested from the inner cheek under direct vision, maintaining a minimum 1 cm margin from the angle of the mouth and Stensen's duct. The graft was defatted and tailored to the required length. For dorsal onlay, the urethra was mobilised off the corpus spongiosum, opened dorsally along the stricture, and the graft was quilted to the underlying corpora cavernosa using interrupted 4-0 polyglactin sutures and anastomosed to the urethral edges. For ventral onlay, the urethra was opened ventrally, and the graft was secured over a 16Fr urethral catheter. In three patients where intraoral harvest was not feasible, a preputial skin graft was used with the same onlay technique. The oral pack was removed on the evening of surgery, and cold liquids were commenced.

Postoperative management and follow-up

Oral antibiotics were continued until catheter removal, which was performed at three to four weeks following a check ascending urethrogram. Patients with prostatomegaly received postoperative alpha-blockers. Postoperative complications were graded using the Clavien-Dindo classification system. Follow-up was conducted at one, three, and six months; IPSS and uroflowmetry with post-void residual urine measurement were recorded at each visit. Failure was defined as the need for any urethral re-intervention due to obstructive symptoms, confirmed by an abnormal urethrogram or urethroscopy. Uroflowmetry was performed as free-flow uroflowmetry without pressure-flow studies. A minimum voided volume of 150 ml was required for a valid Qmax measurement. The highest Qmax value from a minimum of two consecutive voids was recorded at each assessment. Success was defined as Qmax ≥15 ml/second at six months without re-intervention. Uroflowmetry assessments were performed and interpreted by the treating clinical team; independent blinded outcome assessment was not performed, which represents a study limitation.

Statistical analysis

Data were compiled in Microsoft Excel (Microsoft Corporation, Redmond, Washington, United States) and analysed using IBM SPSS Statistics for Windows, version 20.0 (IBM Corp., Armonk, New York, United States) and MedCalc version 19.5 (MedCalc Software Ltd, Ostend, Belgium). Continuous variables are reported as mean ± standard deviation (SD) with range. Categorical variables are reported as frequencies and percentages. Pre- and post-operative IPSS were compared using the Wilcoxon signed-rank test, given the non-normal distribution of ordinal IPSS data. Change in IPSS across multiple postoperative time points was assessed using the Friedman test. Postoperative Qmax values at each follow-up visit were compared against the preoperative threshold of 10 ml/second using a one-sample Wilcoxon signed-rank test; change in Qmax across postoperative time points was additionally assessed using the Friedman test. A p-value of <0.05 was considered statistically significant.

## Results

Patient and operative characteristics

Twenty male patients meeting the inclusion criteria were enrolled over the study period. The mean age of participants was not formally recorded; patients were adults of all age groups, reflecting the typical demographic of urethral stricture disease in the Indian population. All patients had undergone a minimum of two prior OIU procedures: 13 patients (65%) had undergone two prior OIUs, six (30%) had undergone three, and one (5%) had undergone four. The mean duration since the first OIU was 15.30 ± 6.42 months (range 8-36 months); 60% of patients had a duration greater than 12 months since their initial OIU.

Mean preoperative IPSS was 17.90 ± 1.86 (range 14-20), indicating predominantly severe lower urinary tract symptoms. All patients had a preoperative Qmax below 10 ml/second on free-flow uroflowmetry; however, individual preoperative Qmax values were not recorded as discrete data points, representing a study limitation. Mean stricture length was 4.80 ± 1.94 cm (range 2-10 cm); 14 patients (70%) had strictures less than 5 cm in length. Stricture location and degree of spongiofibrosis were not systematically classified. BMG was used in 17 patients (85%); preputial skin graft was used in three patients (15%) in whom intraoral harvest was not feasible due to intraoral pathology, with an otherwise identical surgical technique and onlay configuration. Dorsal onlay was employed in 16 patients (80%); ventral onlay in four (20%). Mean graft length was 7.05 ± 2.24 cm (range 5-14 cm). Mean catheterisation duration was 16.40 ± 3.05 days (range 12-21 days). Patient and operative characteristics are summarised in Table 1.

**Table 1:** Patient and operative characteristics (N=20) OIU: optical internal urethrotomy

Variable		Value
Prior OIU attempts	2	13 (65%)
	3	6 (30%)
	4	1 (5%)
Duration since first OIU (months), mean ± SD (range)		15.30 ± 6.42 (8–36)
Preoperative IPSS, mean ± SD (range)		17.90 ± 1.86 (14–20)
Preoperative Qmax		<10 ml/second (all patients)
Stricture length (cm), mean ± SD (range)		4.80 ± 1.94 (2–10)
Stricture length	<5 cm	14 (70%)
	≥5 cm	6 (30%)
Graft type	BMG	17 (85%)
	Preputial skin	3 (15%)
Graft technique	Dorsal onlay	16 (80%)
	Ventral onlay	4 (20%)
Graft length (cm), mean ± SD (range)		7.05 ± 2.24 (5–14)
Duration of catheterisation (days), mean ± SD (range)		16.40 ± 3.05 (12–21)

Postoperative IPSS

Follow-up was completed by all 20 patients at one month, 19 (95%) at three months, and 18 (90%) at six months. Two patients were lost to follow-up: one after the one-month visit and one after the three-month visit; both had experienced major postoperative complications (Clavien-Dindo Grade 3).

Mean IPSS fell from 17.90 ± 1.86 preoperatively to 3.55 ± 1.70 at one month, 3.37 ± 1.34 at three months, and 3.33 ± 1.19 at six months. The reduction from baseline to six months was statistically significant (mean reduction 14.39 ± 2.15 points; p<0.001, Wilcoxon signed-rank test). All 18 patients with six-month data demonstrated improvement in IPSS relative to their preoperative scores. The Friedman test confirmed a statistically significant progressive change across the three postoperative time points (chi-square = 25.33, df = 2, p<0.001). IPSS data are presented in Table 2.

**Table 2:** Pre- and post-operative IPSS at different timepoints *Wilcoxon signed-rank test IPSS: International Prostate Symptom Score

Timepoint	Number of patients	IPSS, mean ± SD	p-value vs Preoperative*
Preoperative	20	17.90 ± 1.86	—
1 Month	20	3.55 ± 1.70	<0.001
3 Months	19	3.37 ± 1.34	<0.001
6 Months	18	3.33 ± 1.19	<0.001

Postoperative Qmax

All patients had a preoperative Qmax below 10 ml/second. Mean postoperative Qmax was 17.40 ± 3.38 ml/second at one month (range 10-26), 17.79 ± 2.97 ml/second at three months (range 14-26), and 17.33 ± 2.14 ml/second at six months (range 14-22). Postoperative Qmax values at all three time points were significantly above the preoperative threshold of 10 ml/second (p<0.001 at each time point, one-sample Wilcoxon signed-rank test). The Friedman test confirmed a statistically significant change in Qmax across the three postoperative time points (chi-square = 8.24, df = 2, p = 0.016). Qmax data are presented in Table 3.

**Table 3:** Qmax at different timepoints

Timepoint	Number of patients	Qmax (ml/second), mean ± SD	Range (ml/second)
Preoperative	20	<10 (all patients)	—
1 Month	20	17.40 ± 3.38	10–26
3 Months	19	17.79 ± 2.97	14–26
6 Months	18	17.33 ± 2.14	14–22

Success rate

At six months, 17 of 18 patients with available follow-up data (94.4%) achieved the primary success criterion of Qmax ≥15 ml/second without requiring urethral re-intervention. One patient (5.6%) had a Qmax of 14 ml/second at six months and remained under observation without re-intervention at the time of reporting.

Postoperative complications

No complications were recorded in 11 patients (55%). Minor complications not requiring specific intervention beyond standard postoperative care (Grade 1) occurred in four patients (20%). Complications requiring pharmacological treatment (Grade 2) were observed in three patients (15%). Two patients (10%) experienced major complications requiring procedural intervention (Grade 3a, n=1) or operative re-intervention under general anaesthesia (Grade 3b, n=1). Overall, 90% of patients experienced no or minor complications (Grade 0-2). Complication data are presented in Table 4.

**Table 4:** Postoperative complications: Clavien-Dindo classification

Grade	Description	Frequency	Percentage
0	No complication	11	55.0
1	Minor; deviation from normal postoperative course, no intervention	4	20.0
2	Requiring pharmacological treatment	3	15.0
3a	Requiring endoscopic or radiological intervention	1	5.0
3b	Requiring operative intervention under general anaesthesia	1	5.0
Total		20	100.0

## Discussion

Recurrent urethral stricture following failed OIU presents a distinct reconstructive challenge. Repeated endoscopic incisions induce progressive spongiofibrosis, compromise urethral plate vascularity, and render subsequent endoscopic management increasingly ineffective [14]. In this setting, augmentation urethroplasty using BMG has emerged as the preferred definitive intervention [9,15].

The principal findings of the present study are a six-month success rate of 94.4%, a statistically significant reduction in IPSS (mean reduction 14.39 ± 2.15 points; p<0.001), a significant improvement in postoperative Qmax at all follow-up time points (p<0.001), and an acceptable complication profile with 90% of patients experiencing no or minor complications.

Our success rate compares favourably with published series. Javali et al., in their study of 21 patients undergoing redo BMG urethroplasty following previously failed urethroplasty, reported an 85.7% success rate at a mean follow-up of 42.4 months [16]. Our six-month results are at the higher end of this published range, likely reflecting careful patient selection, standardised perioperative protocols, and the relatively shorter follow-up duration.

The dramatic reduction in IPSS, from a mean of 17.90 preoperatively to 3.33 at six months, reflects substantial symptomatic relief consistent with near-complete resolution of obstructive voiding symptoms. The Friedman test confirmed this improvement was progressive and sustained across all three postoperative assessment points (p<0.001), indicating a stable reconstructive outcome rather than a transient postoperative response.

BMG was used in 85% of patients, consistent with current guidelines recommending it as the preferred graft material for anterior urethral reconstruction [17]. The dorsal onlay configuration was employed in 80% of cases. Dorsal fixation to the corpora cavernosa provides a mechanically stable bed that minimises graft sacculation, a recognised complication of ventral placement associated with luminal narrowing over time [18]. Three patients who received preputial grafts, in whom intraoral harvest was not feasible, did not demonstrate substantially different outcomes, consistent with published evidence of comparable short-term results between graft types in experienced hands [19].

Two patients (10%) experienced major complications (Clavien-Dindo Grade 3), both subsequently lost to follow-up. These corresponded to the most surgically complex cases, the longest stricture (10 cm), the greatest number of prior OIUs (four), and the most prolonged interval since initial OIU (36 months), suggesting that disease complexity and prior intervention burden may contribute to complication risk, a hypothesis warranting prospective evaluation in larger series.

The study has several limitations. The sample size of 20 patients from a single tertiary centre limits statistical power and external validity, and a larger multicentre cohort would be required to draw definitive conclusions. Patient age, stricture location, aetiology, and degree of spongiofibrosis were not systematically recorded, precluding subgroup analyses by these variables. Intraoperative selection between dorsal and ventral onlay was based on qualitative rather than standardised objective criteria, limiting reproducibility. Outcome assessments were not performed by blinded independent evaluators, and the absence of a control group prevents direct comparison with alternative approaches. Individual preoperative Qmax values were not recorded as discrete data points, limiting formal paired statistical comparison of uroflowmetric outcomes.

From an analytical standpoint, two patients lost to follow-up after major complications were excluded from the six-month success rate calculation; applying a strict intention-to-treat analysis would yield a success rate of 85% (17/20) rather than 94.4% (17/18), and readers should interpret the reported figure accordingly. The inclusion of three preputial graft recipients alongside 17 BMG patients introduces graft heterogeneity that could not be analysed separately given the small subgroup size. Finally, follow-up was limited to six months; as stricture recurrence frequently occurs within one to five years of surgery, longer follow-up is required to confirm the durability of these results.

## Conclusions

This prospective study demonstrates that augmentation urethroplasty achieves significant short-term symptomatic and functional improvement in patients with recurrent urethral stricture following repeated failed OIU. The results support the early effectiveness of this procedure following failed endoscopic management. The complication profile was acceptable, with the majority of patients experiencing no or minor complications graded as Clavien-Dindo 0-2.

These findings support augmentation urethroplasty as a safe and effective early treatment option following repeated failed OIU, with BMG as the preferred graft material in appropriately selected patients. However, given the small sample size and six-month follow-up duration, these conclusions should be interpreted as preliminary. Prospective multicentre studies with longer follow-up, standardised outcome reporting, and systematic baseline data collection are warranted to confirm long-term durability and define predictors of success in this patient population.
